# Supplementary material for: Exploring the Effect of Mechanical Anisotropy of Protein Structures in the Unfoldase Mechanism of AAA+ Molecular Machines
Source: Nanomaterials (Basel). 2022 May 28;12(11):1849. doi: 10.3390/nano12111849 (PMC9182431; doi:10.3390/nano12111849)
Supplement: Supplementary file 1 [file nanomaterials-12-01849-s001.zip › nanomaterials-1688458-supplementary-SI.pdf]

nanomaterials

MDPI

Supplementary Materials

Exploring the Effect of Mechanical Anisotropy of Protein Structures in the Unfoldase Mechanism of AAA+ Molecular Machines

Rohith Anand Varikoti, Hewafonsekage Yasan Y. Fonseka, Maria S. Kelly, Alex Javidi, Mangesh Damre, Sarah Mullen, Jimmie L. Nugent IV, Christopher M. Gonzales, George Stan, and Ruxandra I. Dima

Force profiles and orientations of spastin machine acting of MT filament

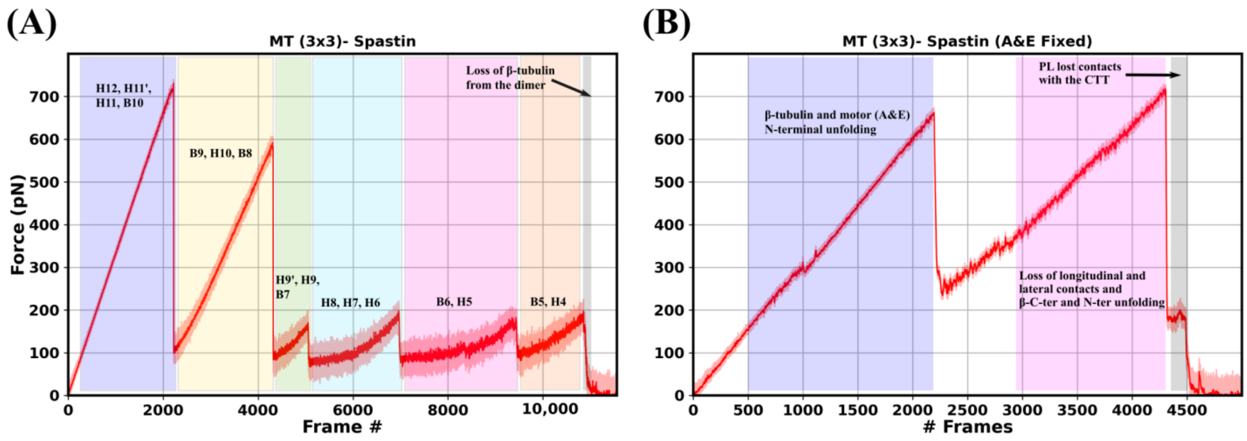

Figure S1. Force vs frame number profile for the action of the spastin hexameric motor on a MT_{3x3} lattice. Here, 1 frame = 0.04 ms. The motor acts by pulling on the C-terminal end of the central β monomer when the N-term residues of (**A**) all spastin chains are fixed, and of (**B**) only spastin chains A and E are fixed.

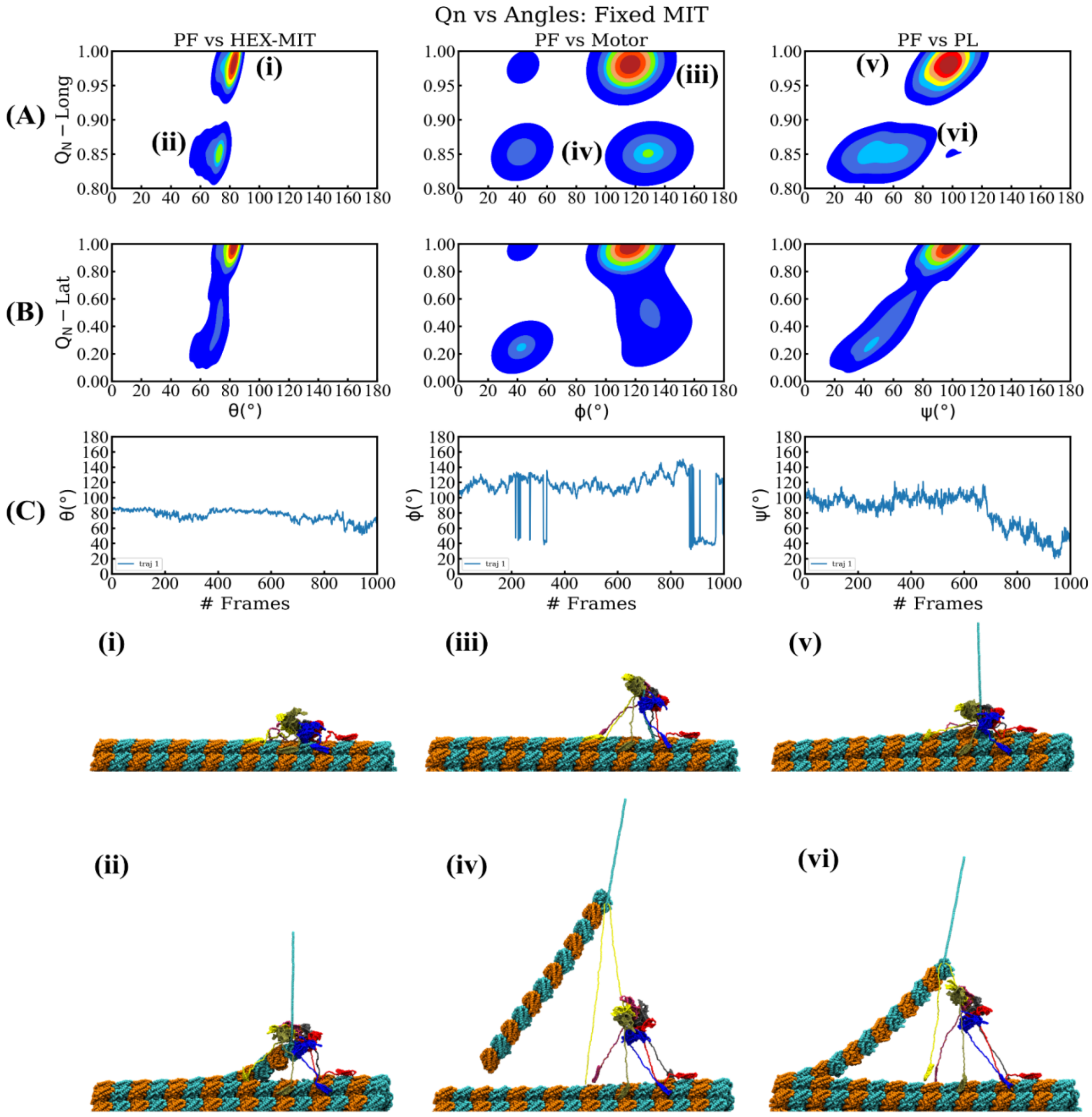

Figure S2. Results of the spastin machine acting on a MT filament when the N-terminal ends of all the MIT domains are fixed on the surface of the MT lattice and the interaction between the spastin machine and the MT lattice is 1.0 kcal/mol. This is similar to Fig. 2 in the main text that depicts the free energy landscape in the plane of fractional loss of longitudinal (**A**) and lateral (**B**) native contacts (Q_N) as well as the time evolution of the three angles from the upper plots versus the simulation frame (**C**). Each column from left to right represents the orientation explained between the pulled protofilament (PF6) and the angle made by the principal axis of the severing enzyme (HEX-MIT), of the motor, and of the central pore loops (PLs). Instantaneous snapshots of the representative structures corresponding to the labeled minima in the free energy plots (**i-vi**) are shown at the bottom.

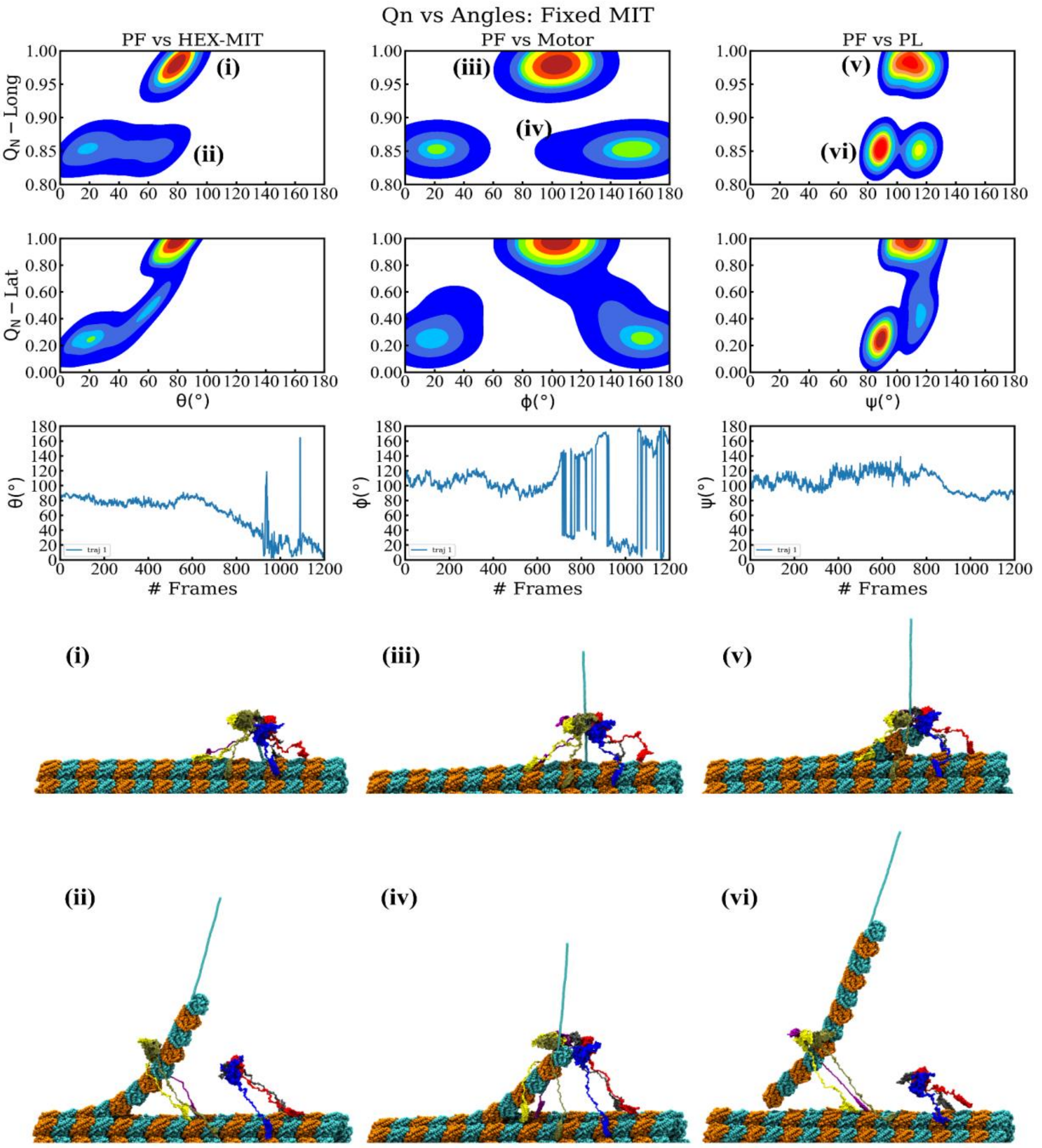

Figure S3. Similar to Fig. S2, while only the contacts between the E15 peptide at the C-terminal end of the pulled tubulin monomer and spastin are set to 1.0 kcal/mol. The free energy landscape in the plane of fractional loss of longitudinal (**A**) and lateral (**B**) native contacts (Q_N) as well as the time evolution of the three angles from the upper plots versus the simulation frame (**C**) is shown above. Each column from left to right represents the orientation explained between the pulled protofilament (PF6) and the angle made by the principal axis of the severing enzyme (HEX-MIT), of the motor, and of the central pore loops (PLs). Instantaneous snapshots of the representative structures corresponding to the labeled minima in the free energy plots (**i-vi**) are shown at the bottom.

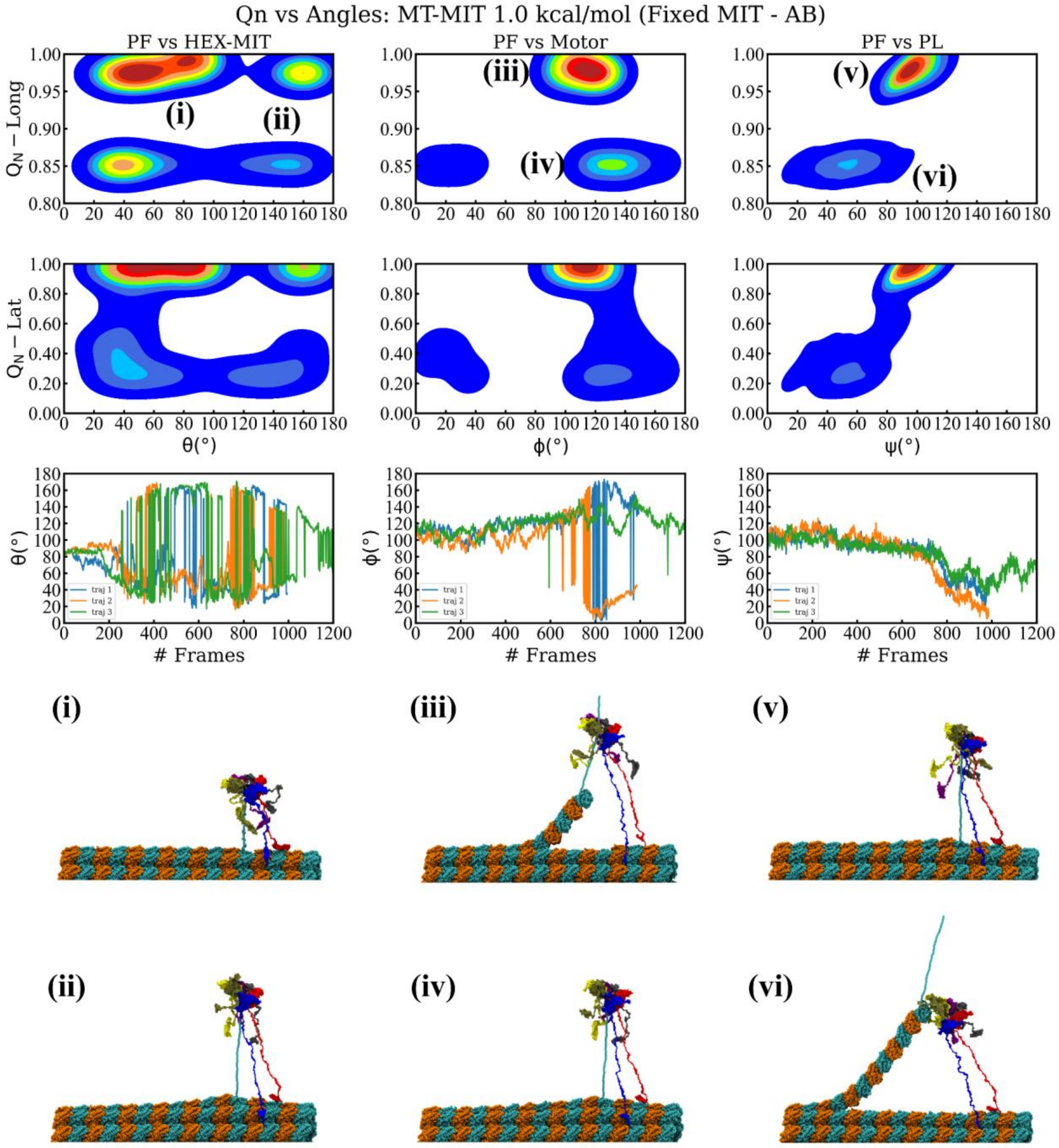

Figure S4. Similar to Fig. S2, but when fixing only the N-terminal ends of the MIT domains from chains A and B in spastin. The free energy landscape in the plane of fractional loss of longitudinal (**A**) and lateral (**B**) native contacts (Q_N) as well as the time evolution of the three angles from the upper plots versus the simulation frame (**C**) is shown above. Each column from left to right represents the orientation explained between the pulled protofilament (PF6) and the angle made by the principal axis of the severing enzyme (HEX-MIT), of the motor, and of the central pore loops (PLs). Instantaneous snapshots of the representative structures corresponding to the labeled minima in the free energy plots (**i-vi**) are shown at the bottom.

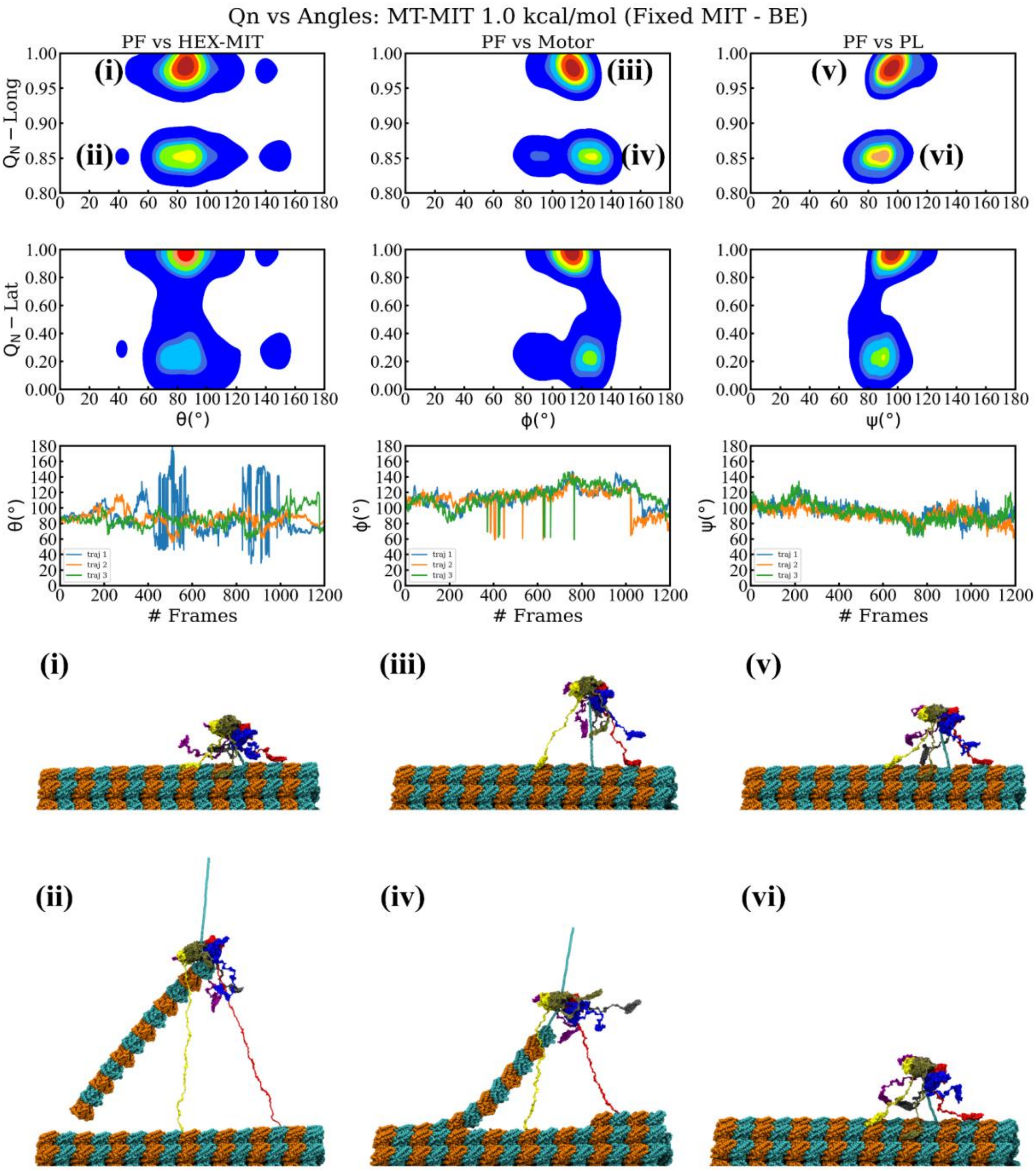

Figure S5. Similar to Fig. S2, but when fixing only the N-terminal ends of the MIT domains from chains B and E in spastin. The free energy landscape in the plane of fractional loss of longitudinal (**A**) and lateral (**B**) native contacts (Q_N) as well as the time evolution of the three angles from the upper plots versus the simulation frame (**C**) is shown above. Each column from left to right represents the orientation explained between the pulled protofilament (PF6) and the angle made by the principal axis of the severing enzyme (HEX-MIT), of the motor, and of the central pore loops (PLs). Instantaneous snapshots of the representative structures corresponding to the labeled minima in the free energy plots (**i-vi**) are shown at the bottom.

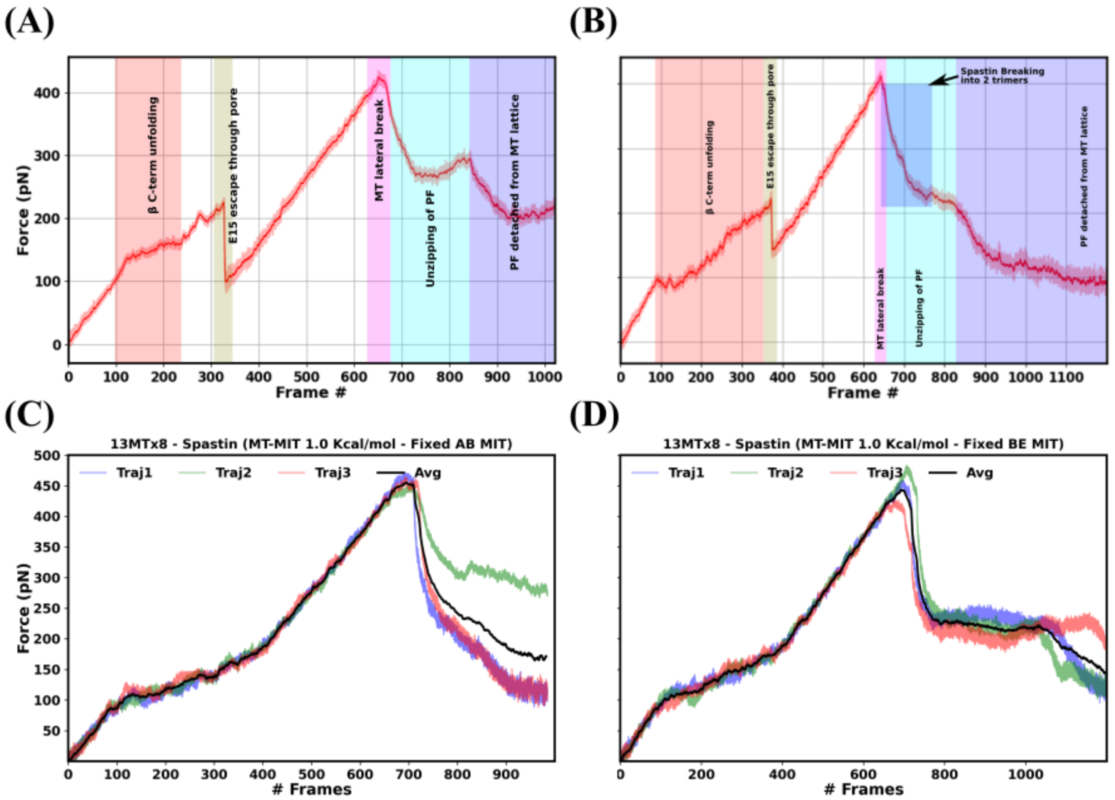

C

Figure S6. Force vs frame number profile for the action of the spastin hexameric machine on a 8 dimers long, 13PF MT lattice when keeping the N-terminal ends (**A**) of all MIT domains fixed on the MT lattice and all contacts between the MT and HEX-MIT are set to 1.0 kcal/mol, (**B**) of all MIT domains fixed on the MT lattice and only the contacts between the MT and MIT, and between the MT and PL are set to 1.0 kcal/mol, (**C**) of only the MIT domains from two consecutive protomers (chains A and B) fixed and only the contacts between the MT and MIT, and between the MT and PL are set to 1.0 kcal/mol, and (**D**) of only the MIT domains from two opposite protomers (chains B and E) fixed and only the contacts between the MT and MIT, and between the MT and PL are set to 1.0 kcal/mol.

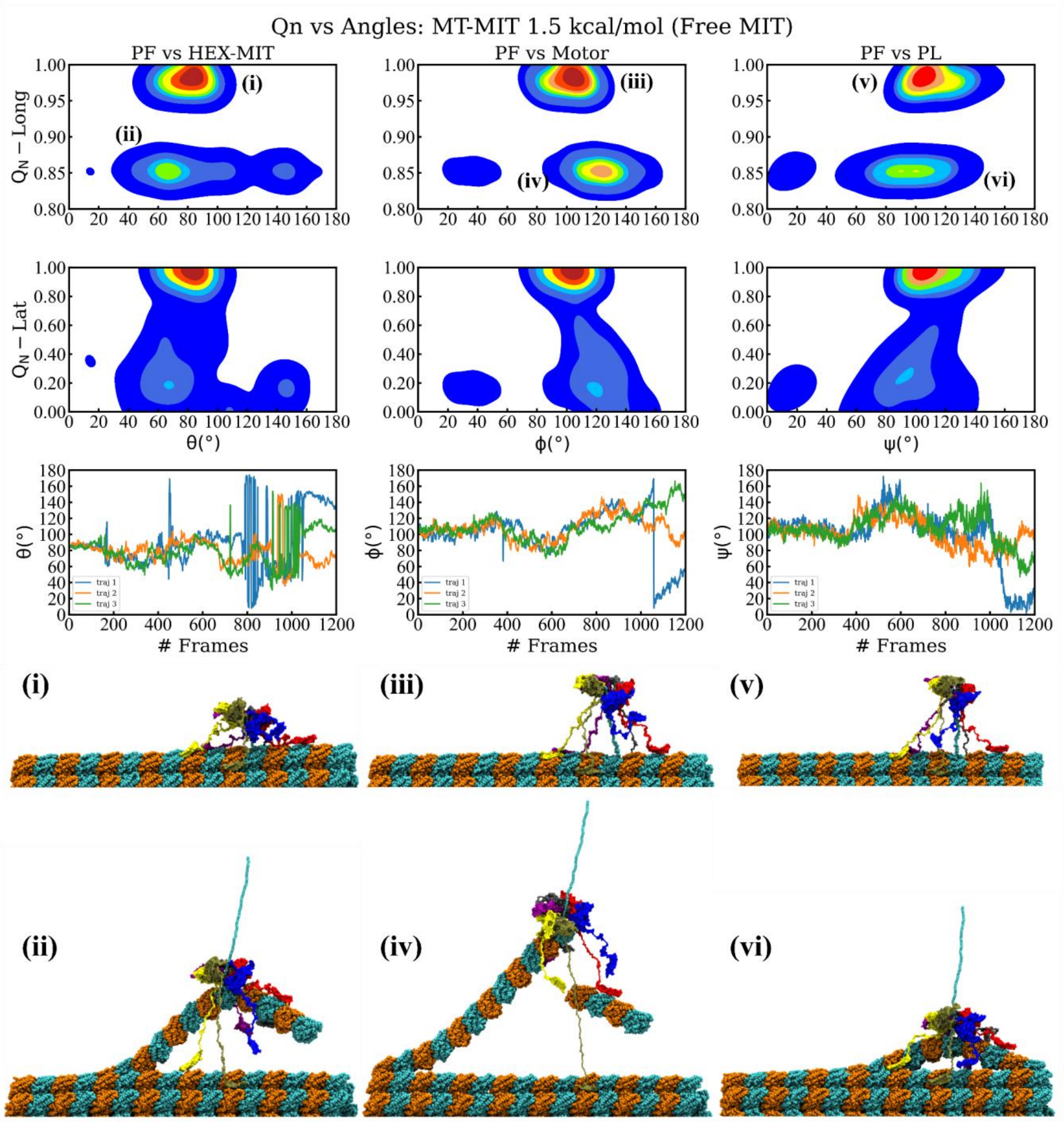

Figure S7. Results of the spastin machine acting on a MT filament for the interaction strength between its MIT domains and the MT lattice set to 1.5 kcal/mol. This is similar to Fig. 2 in the main text. The free energy landscape in the plane of fractional loss of longitudinal (**A**) and lateral (**B**) native contacts (Q_N) as well as the time evolution of the three angles from the upper plots versus the simulation frame (**C**) is shown above. Each column from left to right represents the orientation explained between the pulled protofilament (PF6) and the angle made by the principal axis of the severing enzyme (HEX-MIT), of the motor, and of the central pore loops (PLs). Instantaneous snapshots of the representative structures corresponding to the labeled minima in the free energy plots (**i-vi**) are shown at the bottom.

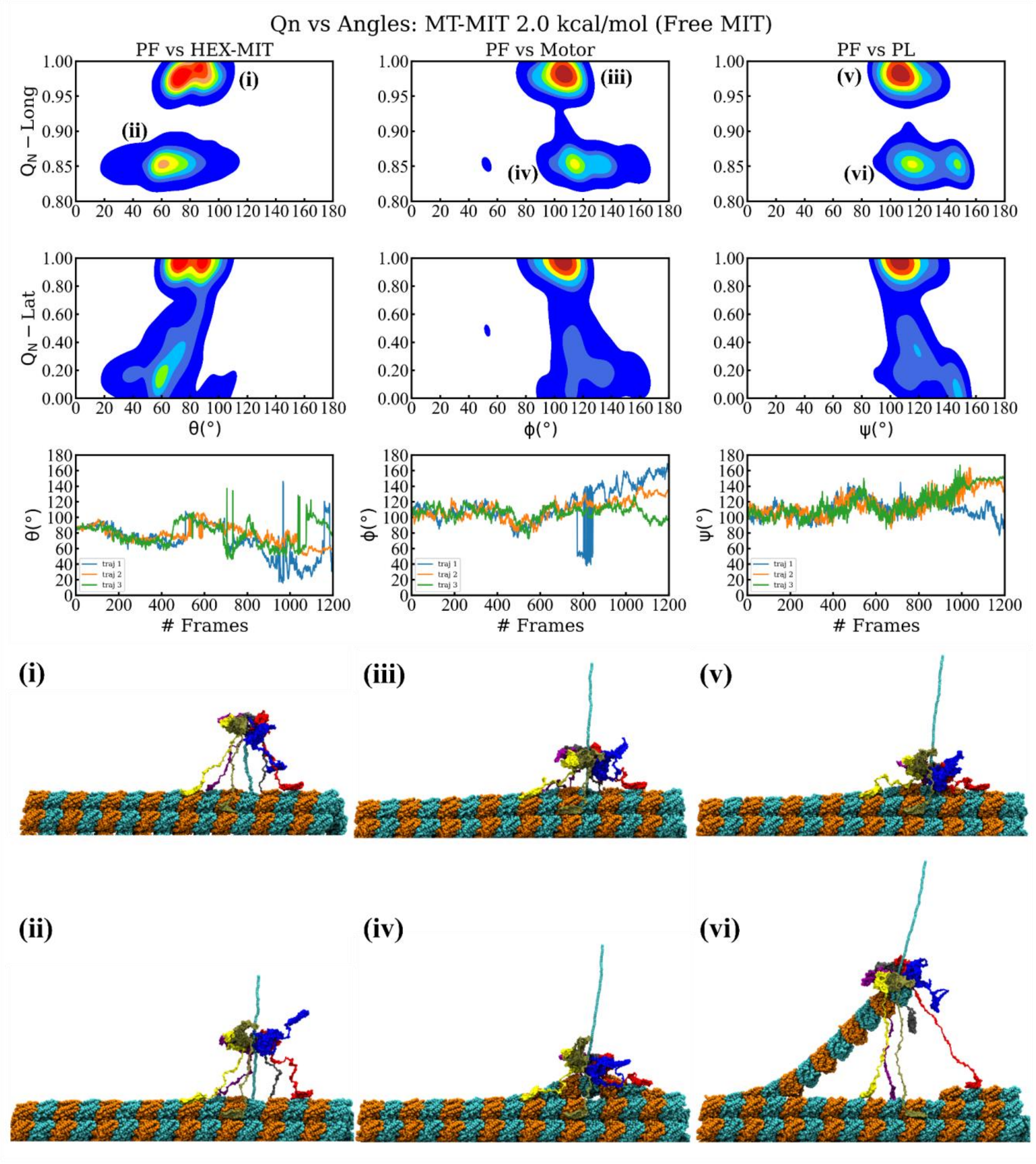

Figure S8. Similar to Fig. S7, for the interaction strength between its MIT domains and the MT lattice set to 2.0 kcal/mol. The free energy landscape in the plane of fractional loss of longitudinal (**A**) and lateral (**B**) native contacts (Q_N) as well as the time evolution of the three angles from the upper plots versus the simulation frame (**C**) is shown above. Each column from left to right represents the orientation explained between the pulled protofilament (PF6) and the angle made by the principal axis of the severing enzyme (HEX-MIT), of the motor, and of the central pore loops (PLs). Instantaneous snapshots of the representative structures corresponding to the labeled minima in the free energy plots (**i-vi**) are shown at the bottom.

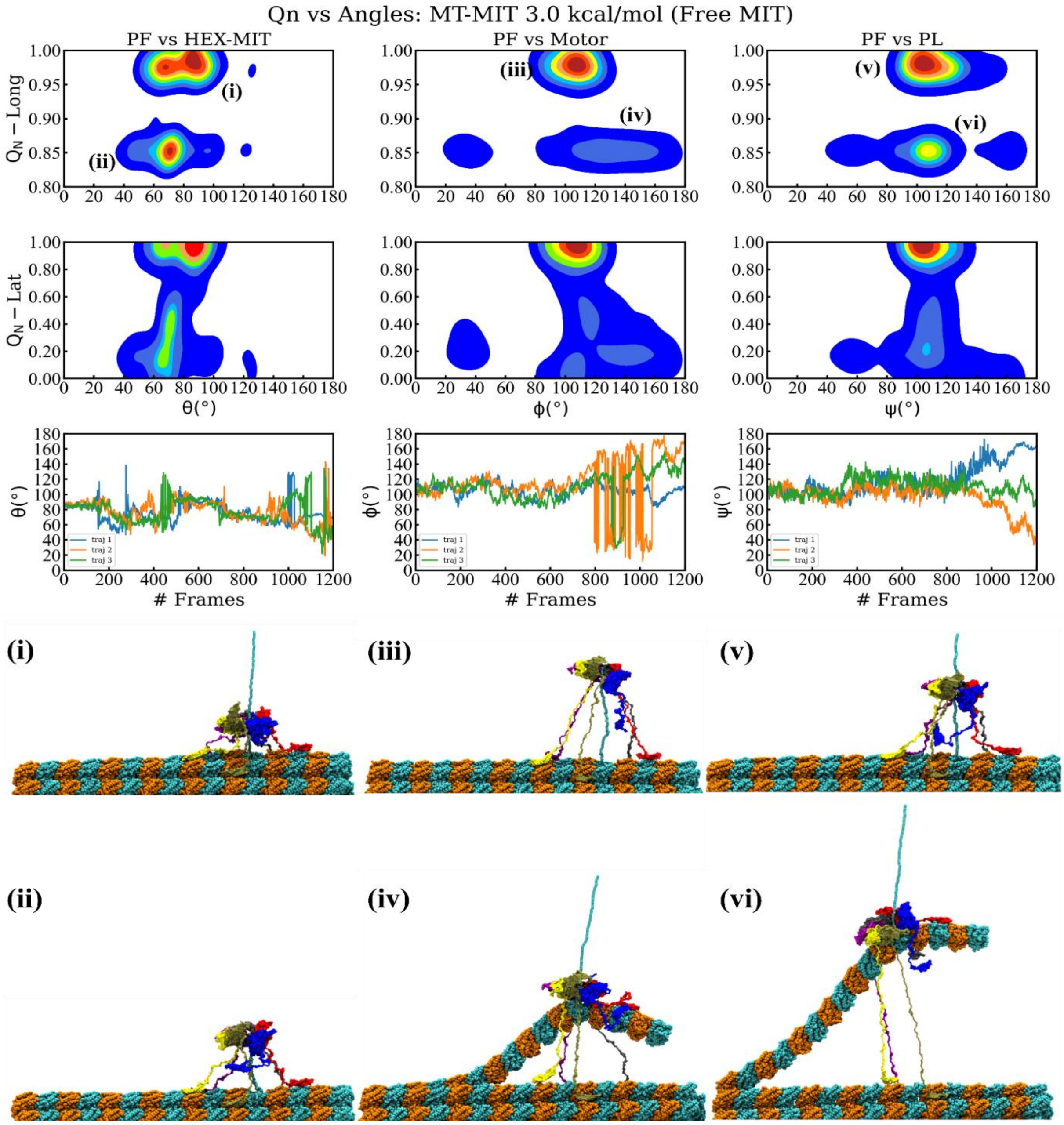

Figure S9. Similar to Fig. S7, for the interaction strength between its MIT domains and the MT lattice set to 3.0 kcal/mol. The free energy landscape in the plane of fractional loss of longitudinal (**A**) and lateral (**B**) native contacts (Q_N) as well as the time evolution of the three angles from the upper plots versus the simulation frame (**C**) is shown above. Each column from left to right represents the orientation explained between the pulled protofilament (PF6) and the angle made by the principal axis of the severing enzyme (HEX-MIT), of the motor, and of the central pore loops (PLs). Instantaneous snapshots of the representative structures corresponding to the labeled minima in the free energy plots (**i-vi**) are shown at the bottom.

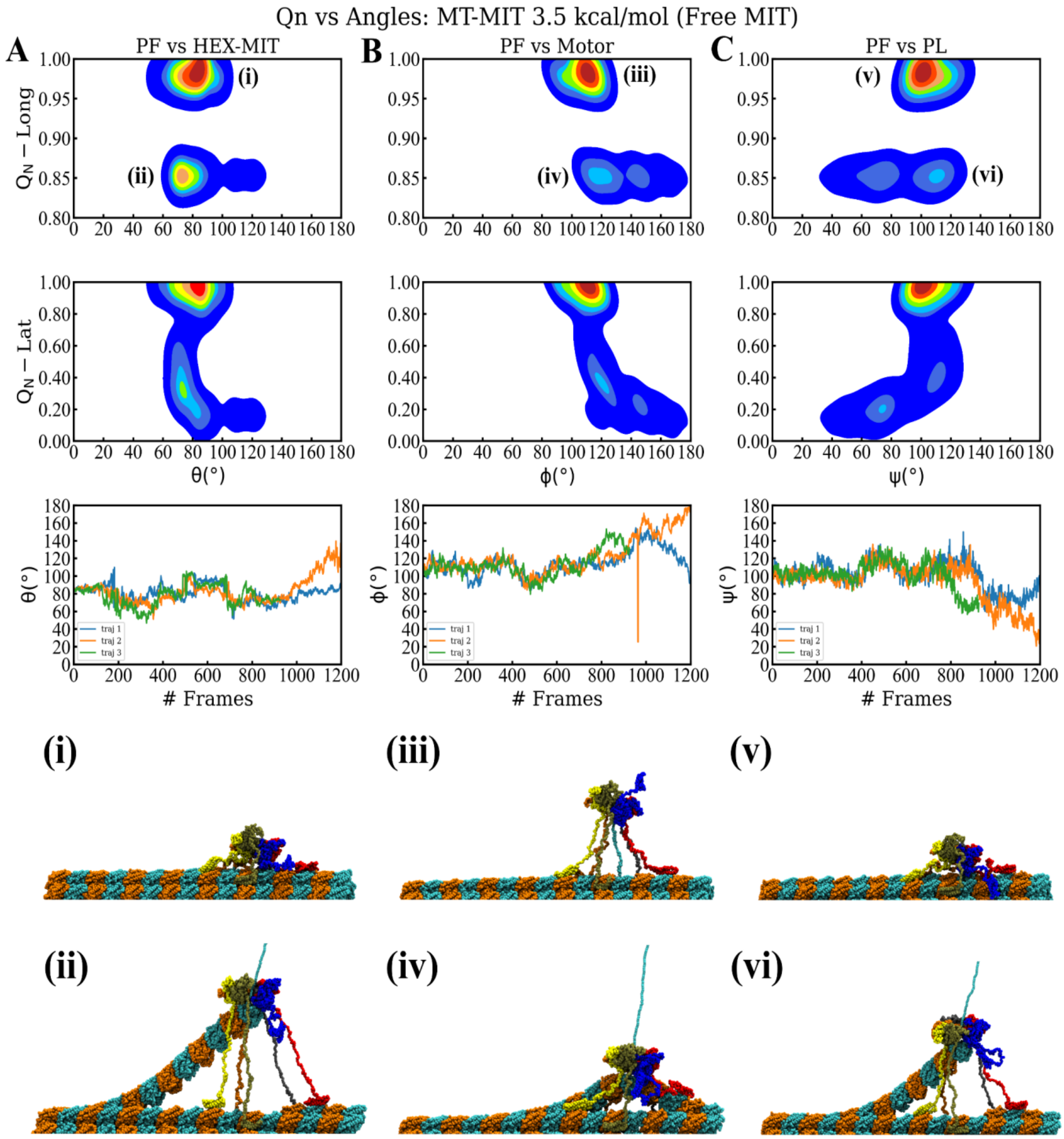

Figure S10. Similar to Fig. S7, for the interaction strength between its MIT domains and the MT lattice set to 3.5 kcal/mol. The free energy landscape in the plane of fractional loss of longitudinal (**A**) and lateral (**B**) native contacts (Q_N) as well as the time evolution of the three angles from the upper plots versus the simulation frame (**C**) is shown above. Each column from left to right represents the orientation explained between the pulled protofilament (PF6) and the angle made by the principal axis of the severing enzyme (HEX-MIT), of the motor, and of the central pore loops (PLs). Instantaneous snapshots of the representative structures corresponding to the labeled minima in the free energy plots (**i-vi**) are shown at the bottom.

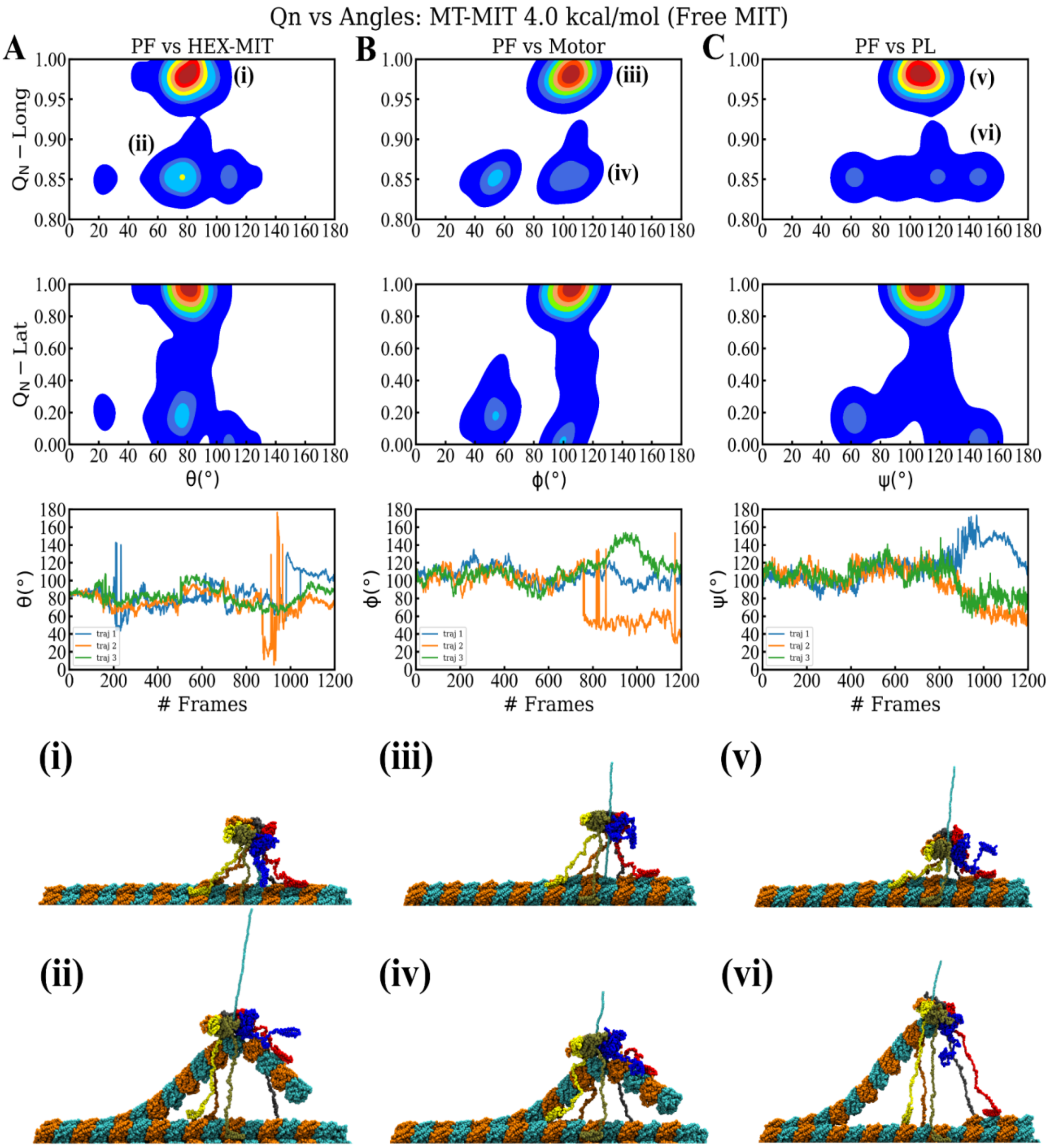

Figure S11. Similar to Fig. S7, for the interaction strength between its MIT domains and the MT lattice set to 4.0 kcal/mol. The free energy landscape in the plane of fractional loss of longitudinal (**A**) and lateral (**B**) native contacts (Q_N) as well as the time evolution of the three angles from the upper plots versus the simulation frame (**C**) is shown above. Each column from left to right represents the orientation explained between the pulled protofilament (PF6) and the angle made by the principal axis of the severing enzyme (HEX-MIT), of the motor, and of the central pore loops (PLs). Instantaneous snapshots of the representative structures corresponding to the labeled minima in the free energy plots (**i-vi**) are shown at the bottom.

Clustering of DHFR conformations and orientations in ClpY–mediated unfolding and translocation pathways

Agglomerative hierarchical clustering with complete linkage (top panel) was used to identify the principal conformations and orientations of WT-DHFR and its CP variants along the ClpY-mediated unfolding and translocation pathways. We used the Euclidean metric to evaluate datasets combining the fraction of native contacts Q_N and the polar angle θ. We computed the appropriate cluster numbers, 5 for each setup, using the silhouette[1], Calinski–Harabasz[2], Davies–Bouldin[3] scores.

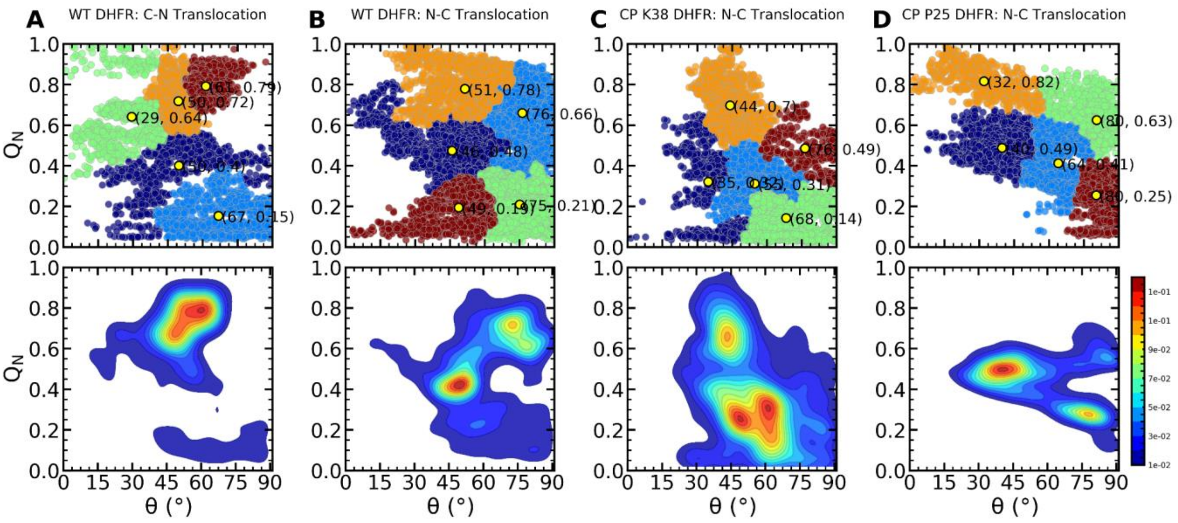

Figure S12. Clustering analysis of DHFR conformations and orientations in ClpY-mediated unfolding and translocation pathways. Principal clusters and probability density maps of datasets of Q_N and the polar angle θ in pathways corresponding to the (**A**)-(**B**) WT-DHFR in (**A**) C-N and (**B**) N-C translocation, (**C**) CP K38 and (**D**) CP P25. Centroids of clusters are indicated using yellow dots.

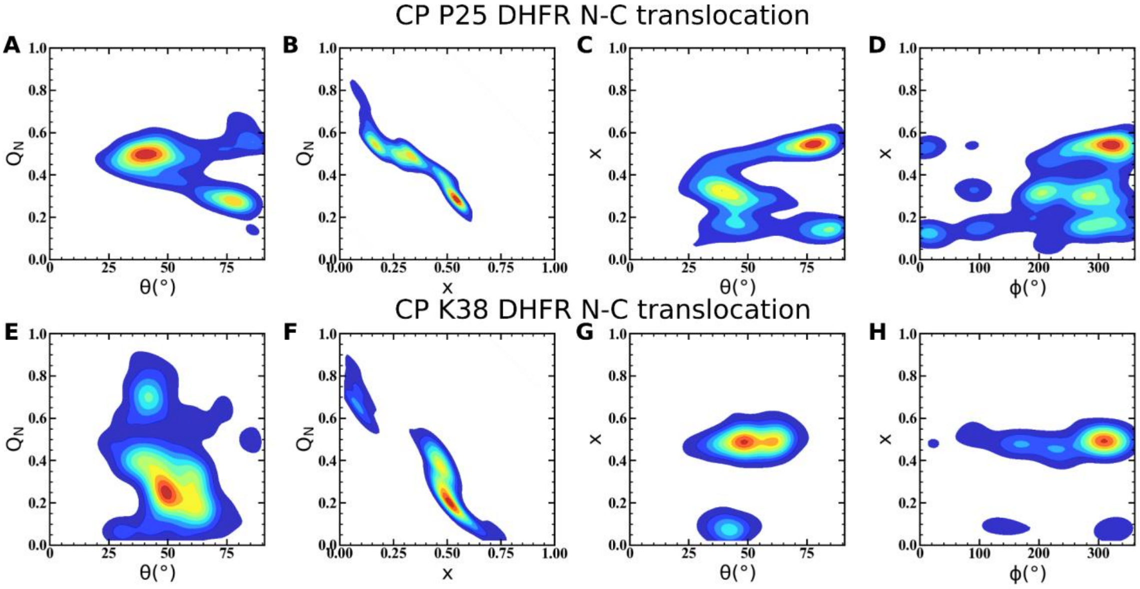

Figure S13. CP DHFR variant orientation at the ClpY pore lumen in unfolding and translocation pathways. Probability density maps of the (**A**) fraction of native contacts Q_N vs. polar angle θ and (**B**) translocation fraction x; (**C**) translocation fraction vs. polar θ and azimuthal φ angles in CP P25 translocation. (**E**)-(**H**) Same as in (**A**)-(**D**) in the CP K38.

References

(1) Rousseeuw, P. J. Silhouettes: a graphical aid to the interpretation and validation of cluster analysis. *J. Comput. Appl. Math.* 1987, 20, 53–65.

(2) Caliński, T.; Harabasz, J. A dendrite method for cluster analysis. *Commun. Stat. Theory Methods* 1974, 3, 1–27.

(3) Davies, D. L.; Bouldin, D. W. A cluster separation measure. *IEEE Trans. Pattern Anal. Mach. Intell.* 1979, 224–227.
